# 2-(3,4-Dimethyl-5,5-dioxo-2*H*,4*H*-pyrazolo[4,3-*c*][1,2]benzothia­zin-2-yl)-*N*′-(2-thienylmethyl­idene)acetohydrazide

**DOI:** 10.1107/S1600536810015084

**Published:** 2010-05-08

**Authors:** Matloob Ahmad, Hamid Latif Siddiqui, Altaf Hussain Khan, Masood Parvez

**Affiliations:** a Applied Chemistry Research Centre, PCSIR Laboratories Complex, Lahore 54600, Pakistan; b Institute of Chemistry, University of the Punjab, Lahore 54590, Pakistan; c Department of Chemistry, The University of Calgary, 2500 University Drive NW, Calgary, Alberta, Canada T2N 1N4

## Abstract

In the title mol­ecule, C_18_H_17_N_5_O_3_S_2_, the heterocyclic thia­zine ring adopts a twist boat conformation, with the S and N atoms displaced by 0.480 (7) and 0.205 (8) Å, respectively, on opposite sides of the mean plane formed by the remaining ring atoms. The pyrazole and benzene rings are tilted at an angle of 10.9 (2)° with respect to one another. The crystal structure is stabilized by inter­molecular N—H⋯O and C—H⋯N hydrogen bonds, resulting in dimers forming nine-membered rings of graph-set motif *R*_2_^2^(9). In addition, inter­molecular C—H⋯O inter­actions result in chains of mol­ecules along the *c* axis, further consolidating the crystal packing.

## Related literature

For the use of 1,2-benzothia­zine derivatives as anti-inflammatory drugs, see: Lombardino *et al.* (1973 ▶); Zia-ur-Rehman *et al.* (2006 ▶). For the synthesis of benzothia­zine derivatives, see: Ahmad *et al.* (2010 ▶). For related structures, see: Ahmad *et al.* (2010 ▶). For graph-set notation, see: Bernstein *et al.* (1994 ▶).
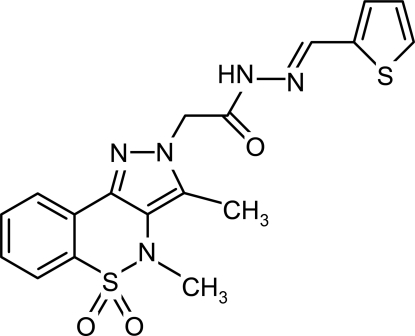


## Experimental

### 

#### Crystal data

C_18_H_17_N_5_O_3_S_2_
*M* *_r_* = 415.49
Orthorhombic, 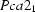

*a* = 18.5474 (2) Å
*b* = 11.6670 (5) Å
*c* = 8.4783 (7) Å
*V* = 1834.64 (17) Å^3^
*Z* = 4
Mo *K*α radiation
μ = 0.32 mm^−1^
*T* = 173 K
0.28 × 0.06 × 0.04 mm

#### Data collection

Nonius KappaCCD diffractometer
Absorption correction: multi-scan (*SORTAV*; Blessing, 1997 ▶) *T*_min_ = 0.915, *T*_max_ = 0.987
3078 measured reflections
1725 independent reflections
1623 reflections with *I* > 2σ(*I*)
*R*_int_ = 0.028

#### Refinement

*R*[*F* ^2^ > 2σ(*F* ^2^)] = 0.036
*wR*(*F* ^2^) = 0.087
*S* = 1.09
1725 reflections
255 parameters
1 restraint
H-atom parameters constrained
Δρ_max_ = 0.21 e Å^−3^
Δρ_min_ = −0.20 e Å^−3^

### 

Data collection: *COLLECT* (Hooft, 1998 ▶); cell refinement: *DENZO* (Otwinowski & Minor, 1997 ▶); data reduction: *SCALEPACK* (Otwinowski & Minor, 1997 ▶); program(s) used to solve structure: *SHELXS97* (Sheldrick, 2008 ▶); program(s) used to refine structure: *SHELXL97* (Sheldrick, 2008 ▶); molecular graphics: *ORTEP-3 for Windows* (Farrugia, 1997 ▶); software used to prepare material for publication: *SHELXL97*.

## Supplementary Material

Crystal structure: contains datablocks global, I. DOI: 10.1107/S1600536810015084/jh2146sup1.cif

Structure factors: contains datablocks I. DOI: 10.1107/S1600536810015084/jh2146Isup2.hkl

Additional supplementary materials:  crystallographic information; 3D view; checkCIF report

## Figures and Tables

**Table 1:** Hydrogen-bond geometry (Å, °)

*D*—H⋯*A*	*D*—H	H⋯*A*	*D*⋯*A*	*D*—H⋯*A*
N4—H4*N*⋯O3^i^	0.88	2.10	2.964 (4)	166
C12—H12*A*⋯N5^i^	0.99	2.52	3.488 (5)	164
C11—H11*B*⋯O1^ii^	0.98	2.40	3.243 (6)	143

Symmetry codes: (i) 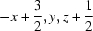; (ii) 
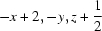
.

**Fig. 1.**
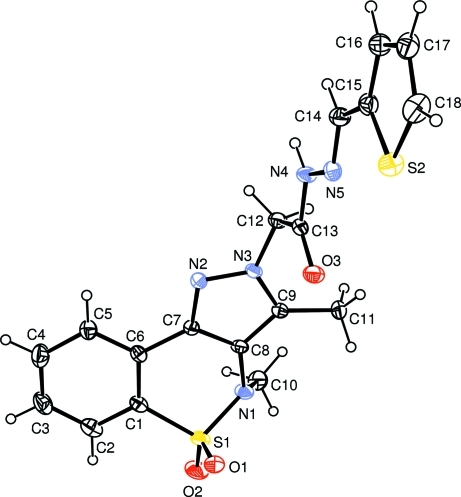
The title molecule plotted with the displacement ellipsoids at 30% probability level (Farrugia, 1997).

**Fig. 2.**
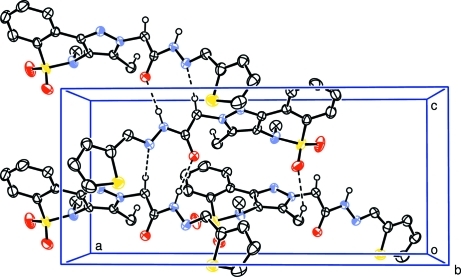
A part of the unit cell showing intermolecular hydrogen bonds by dashed lines; the H-atoms not involved in H-bonds have been excluded for clarity.

## supplementary crystallographic information

### Comment

Benzothiazines represent a class of heterocyclic compounds which exhibit a diverse range of biological activities like anti-inflammatory (Lombardino *et al.*, 1973), antibacterial (Zia-ur-Rehman *et al.*, 2006), etc. Continuing our research on the synthesis of potential biologically active derivatives of benzothiazines, we have fused a pyrazole ring with benzothiazine nucleus and the resulting compounds were found to be potent antioxidants (Ahmad *et al.*, 2010). Herein we report the synthesis and crystal structure of the title compound.

In the title compound (Fig. 1), the bond distances and angles agree with the cortresponding bond distances and angles reported in a closely related compound (Ahmad *et al.*, 2010). The heterocyclic thiazine ring adopts a twist boat conformation with atoms S1 and N1 displaced by 0.480 (7) and 0.205 (8) Å on the opposite sides from the mean planes formed by the remaining ring atoms. The pyrazol and phenyl rings are tilted at 10.9 (2)° with respect to each other. The atoms (S2/O3/N4/N5/C12—C18) of the thiophenylmethylideneacetohydrazide moiety are almost planar with meaximum deviation being 0.070 (3) Å for N4.

The structure is stabilized by intermolecular hydrogen bonds N4—H4N···O3 and C12—H12A···N5, resulting in dimers forming nine membered rings in R_2_^2^(9) graph set motif (Bernstein *et al.*, 1994). In addition, intermolecular interactions C12—H12B···O1 leading to chains of molecules along the *c*-axis further consolidate the crystal packing; the details of hydrogen bonding geometry have been provided in Tab. 1 and Fig. 2.

### Experimental

A mixture of 2-(3,4-dimethyl-5,5-dioxidopyrazolo[4,3-c][1,2]benzothiazin -2(4*H*)-yl)acetohydrazide (10 mmoles), 2-formyl thiophene (12 mmoles), methanol (50 ml) and 2 drops of *o*-phosphoric acid were subjected to reflux for 3 hours. The precipitates formed were collected and washed with methanol. Crystals suitable for XRD were grown in dimethylformamide. mp. 556-557 K, MS m/z: 415.0(M+).

### Refinement

The absolute structure parameter  indicated the presence of racemic twinning. Therefore, a TWIN instruction with the default matrix R = (-1 0 0 , 0 -1 0, 0 0 -1) and a BASF with one parameter was used in the final round of least-squares refinement cycles. Though all the H atoms could be distinguished in the difference Fourier map the H-atoms were included at geometrically idealized positions and refined in riding-model approximation with N—H = 0.88 Å and C—H = 0.95, 0.98 and 0.99 Å for aryl, methy and methylene H-atoms, respectively. The *U*_iso_(H) were allowed at 1.2*U*_eq_(N/C). The final difference map was essentially featurless. 1343 Friedel pairs were merged.

### Crystal data

**Table:**

C_18_H_17_N_5_O_3_S_2_	*F*(000) = 864
*M**_r_* = 415.49	*D*_x_ = 1.504 Mg m^−^^3^
Orthorhombic, *P**c**a*2_1_	Mo *K*α radiation, λ = 0.71073 Å
Hall symbol: P 2c -2ac	Cell parameters from 2348 reflections
*a* = 18.5474 (2) Å	θ = 1.0–27.5°
*b* = 11.6670 (5) Å	µ = 0.32 mm^−^^1^
*c* = 8.4783 (7) Å	*T* = 173 K
*V* = 1834.64 (17) Å^3^	Needle, colorless
*Z* = 4	0.28 × 0.06 × 0.04 mm

### Data collection

**Table:**

Nonius KappaCCD diffractometer	1725 independent reflections
Radiation source: fine-focus sealed tube	1623 reflections with *I* > 2σ(*I*)
graphite	*R*_int_ = 0.028
ω and φ scans	θ_max_ = 25.0°, θ_min_ = 2.1°
Absorption correction: multi-scan (*SORTAV*; Blessing, 1997)	*h* = −22→21
*T*_min_ = 0.915, *T*_max_ = 0.987	*k* = −13→13
3078 measured reflections	*l* = −10→10

### Refinement

**Table:**

Refinement on *F*^2^	Primary atom site location: structure-invariant direct methods
Least-squares matrix: full	Secondary atom site location: difference Fourier map
*R*[*F*^2^ > 2σ(*F*^2^)] = 0.036	Hydrogen site location: difference Fourier map
*wR*(*F*^2^) = 0.087	H-atom parameters constrained
*S* = 1.09	*w* = 1/[σ^2^(*F*_o_^2^) + (0.0336*P*)^2^ + 1.3682*P*] where *P* = (*F*_o_^2^ + 2*F*_c_^2^)/3
1725 reflections	(Δ/σ)_max_ = 0.001
255 parameters	Δρ_max_ = 0.21 e Å^−^^3^
1 restraint	Δρ_min_ = −0.20 e Å^−^^3^

### Special details

**Table:**

Geometry. All esds (except the esd in the dihedral angle between two l.s. planes) are estimated using the full covariance matrix. The cell esds are taken into account individually in the estimation of esds in distances, angles and torsion angles; correlations between esds in cell parameters are only used when they are defined by crystal symmetry. An approximate (isotropic) treatment of cell esds is used for estimating esds involving l.s. planes.
Refinement. Refinement of *F*^2^ against ALL reflections. The weighted *R*-factor wR and goodness of fit *S* are based on *F*^2^, conventional *R*-factors *R* are based on *F*, with *F* set to zero for negative *F*^2^. The threshold expression of *F*^2^ > σ(*F*^2^) is used only for calculating *R*-factors(gt) etc. and is not relevant to the choice of reflections for refinement. *R*-factors based on *F*^2^ are statistically about twice as large as those based on *F*, and *R*- factors based on ALL data will be even larger.

### Fractional atomic coordinates and isotropic or equivalent isotropic displacement parameters (Å^2^)

**Table:**

	*x*	*y*	*z*	*U*_iso_*/*U*_eq_	
S1	1.12931 (5)	0.06283 (9)	0.20843 (14)	0.0366 (3)	
S2	0.61859 (6)	0.54119 (10)	−0.03840 (15)	0.0429 (3)	
O1	1.11825 (16)	0.1277 (3)	0.0679 (4)	0.0451 (8)	
O2	1.18427 (15)	−0.0233 (3)	0.2123 (5)	0.0547 (9)	
O3	0.82031 (14)	0.2742 (3)	0.1289 (3)	0.0375 (7)	
N1	1.05269 (16)	0.0005 (3)	0.2551 (4)	0.0344 (8)	
N2	0.95284 (16)	0.2366 (3)	0.4250 (4)	0.0277 (7)	
N3	0.90015 (15)	0.1669 (3)	0.3686 (4)	0.0270 (7)	
N4	0.73543 (15)	0.3288 (3)	0.3100 (4)	0.0312 (7)	
H4N	0.7209	0.3250	0.4087	0.037*	
N5	0.69763 (15)	0.3935 (3)	0.2017 (4)	0.0316 (7)	
C1	1.1446 (2)	0.1638 (3)	0.3617 (5)	0.0297 (9)	
C2	1.2143 (2)	0.1880 (4)	0.4090 (5)	0.0420 (11)	
H2	1.2537	0.1451	0.3681	0.050*	
C3	1.2262 (2)	0.2749 (4)	0.5164 (6)	0.0469 (12)	
H3	1.2738	0.2905	0.5514	0.056*	
C4	1.1699 (3)	0.3386 (4)	0.5728 (6)	0.0464 (11)	
H4	1.1791	0.4012	0.6416	0.056*	
C5	1.0993 (2)	0.3125 (3)	0.5303 (5)	0.0338 (9)	
H5	1.0603	0.3557	0.5724	0.041*	
C6	1.08596 (19)	0.2230 (3)	0.4259 (5)	0.0262 (8)	
C7	1.01335 (19)	0.1826 (3)	0.3855 (5)	0.0241 (8)	
C8	0.99971 (19)	0.0813 (3)	0.3046 (5)	0.0274 (8)	
C9	0.92568 (19)	0.0723 (3)	0.2924 (5)	0.0286 (8)	
C10	1.0535 (2)	−0.1124 (3)	0.3329 (6)	0.0417 (10)	
H10A	1.0039	−0.1374	0.3526	0.050*	
H10B	1.0795	−0.1067	0.4332	0.050*	
H10C	1.0777	−0.1681	0.2645	0.050*	
C11	0.8794 (2)	−0.0162 (4)	0.2176 (6)	0.0392 (10)	
H11A	0.8362	0.0202	0.1737	0.047*	
H11B	0.8651	−0.0730	0.2969	0.047*	
H11C	0.9062	−0.0542	0.1329	0.047*	
C12	0.8253 (2)	0.1981 (3)	0.3944 (5)	0.0317 (9)	
H12A	0.8214	0.2405	0.4952	0.038*	
H12B	0.7961	0.1275	0.4036	0.038*	
C13	0.79532 (19)	0.2713 (3)	0.2631 (5)	0.0289 (8)	
C14	0.6402 (2)	0.4406 (3)	0.2547 (5)	0.0339 (9)	
H14	0.6268	0.4295	0.3618	0.041*	
C15	0.5956 (2)	0.5102 (4)	0.1539 (5)	0.0363 (10)	
C16	0.5327 (2)	0.5660 (3)	0.1956 (6)	0.0383 (9)	
H16	0.5112	0.5597	0.2968	0.046*	
C17	0.5039 (3)	0.6333 (4)	0.0727 (6)	0.0430 (11)	
H17	0.4608	0.6771	0.0813	0.052*	
C18	0.5445 (3)	0.6279 (4)	−0.0572 (6)	0.0487 (12)	
H18	0.5333	0.6683	−0.1513	0.058*	

### Atomic displacement parameters (Å^2^)

**Table:**

	*U*^11^	*U*^22^	*U*^33^	*U*^12^	*U*^13^	*U*^23^
S1	0.0265 (4)	0.0410 (5)	0.0423 (6)	0.0017 (4)	0.0109 (5)	−0.0047 (6)
S2	0.0460 (6)	0.0429 (6)	0.0398 (6)	0.0039 (5)	0.0011 (6)	0.0019 (5)
O1	0.0430 (17)	0.058 (2)	0.0343 (16)	−0.0118 (15)	0.0114 (15)	−0.0049 (16)
O2	0.0324 (14)	0.0526 (18)	0.079 (3)	0.0093 (13)	0.0159 (19)	−0.014 (2)
O3	0.0276 (14)	0.0575 (19)	0.0273 (16)	0.0057 (13)	0.0015 (13)	0.0031 (14)
N1	0.0298 (16)	0.0280 (16)	0.045 (2)	0.0026 (13)	0.0101 (16)	−0.0056 (16)
N2	0.0242 (15)	0.0309 (16)	0.0280 (18)	0.0007 (13)	−0.0002 (14)	−0.0015 (14)
N3	0.0185 (14)	0.0359 (18)	0.0265 (17)	0.0010 (13)	0.0034 (13)	0.0011 (15)
N4	0.0256 (16)	0.0452 (18)	0.0229 (16)	0.0053 (14)	0.0010 (14)	0.0038 (16)
N5	0.0274 (14)	0.0374 (17)	0.0299 (16)	0.0013 (13)	−0.0050 (16)	0.0003 (17)
C1	0.0237 (18)	0.032 (2)	0.033 (2)	−0.0018 (16)	0.0024 (17)	0.0068 (18)
C2	0.031 (2)	0.051 (3)	0.045 (3)	0.0018 (19)	0.004 (2)	0.011 (2)
C3	0.032 (2)	0.064 (3)	0.045 (3)	−0.010 (2)	−0.010 (2)	0.008 (3)
C4	0.051 (3)	0.049 (3)	0.039 (2)	−0.012 (2)	−0.011 (2)	−0.002 (2)
C5	0.033 (2)	0.034 (2)	0.034 (2)	−0.0001 (17)	−0.0053 (19)	−0.0015 (19)
C6	0.0256 (17)	0.0259 (17)	0.027 (2)	−0.0033 (15)	−0.0010 (16)	0.0076 (16)
C7	0.0228 (17)	0.0236 (17)	0.0259 (19)	0.0010 (14)	0.0008 (15)	−0.0002 (16)
C8	0.0234 (17)	0.0306 (19)	0.028 (2)	0.0011 (14)	0.0084 (17)	0.0001 (17)
C9	0.0262 (18)	0.033 (2)	0.026 (2)	−0.0047 (16)	0.0031 (17)	0.0005 (17)
C10	0.049 (2)	0.029 (2)	0.047 (3)	−0.0031 (19)	0.006 (2)	−0.001 (2)
C11	0.035 (2)	0.048 (2)	0.034 (2)	−0.0156 (18)	0.001 (2)	−0.008 (2)
C12	0.0240 (18)	0.046 (2)	0.0252 (19)	0.0005 (17)	0.0027 (16)	0.0000 (19)
C13	0.0200 (17)	0.038 (2)	0.029 (2)	−0.0025 (16)	−0.0028 (17)	−0.0026 (17)
C14	0.0309 (19)	0.037 (2)	0.034 (2)	0.0050 (17)	0.0031 (18)	−0.0022 (18)
C15	0.031 (2)	0.033 (2)	0.045 (3)	0.0012 (17)	−0.0037 (19)	−0.0070 (19)
C16	0.0341 (19)	0.038 (2)	0.043 (2)	0.0047 (17)	−0.003 (2)	−0.005 (2)
C17	0.046 (2)	0.037 (2)	0.046 (3)	0.0079 (19)	−0.011 (2)	−0.006 (2)
C18	0.062 (3)	0.038 (2)	0.046 (3)	0.016 (2)	−0.012 (3)	0.003 (2)

### Geometric parameters (Å, °)

**Table:**

S1—O1	1.426 (4)	C4—H4	0.9500
S1—O2	1.432 (3)	C5—C6	1.391 (5)
S1—N1	1.645 (3)	C5—H5	0.9500
S1—C1	1.777 (4)	C6—C7	1.467 (5)
S2—C18	1.714 (4)	C7—C8	1.390 (5)
S2—C15	1.724 (5)	C8—C9	1.381 (5)
O3—C13	1.229 (5)	C9—C11	1.485 (5)
N1—C8	1.425 (5)	C10—H10A	0.9800
N1—C10	1.472 (5)	C10—H10B	0.9800
N2—C7	1.329 (5)	C10—H10C	0.9800
N2—N3	1.358 (4)	C11—H11A	0.9800
N3—C9	1.364 (5)	C11—H11B	0.9800
N3—C12	1.451 (5)	C11—H11C	0.9800
N4—C13	1.358 (5)	C12—C13	1.509 (6)
N4—N5	1.380 (4)	C12—H12A	0.9900
N4—H4N	0.8800	C12—H12B	0.9900
N5—C14	1.280 (5)	C14—C15	1.441 (6)
C1—C2	1.384 (6)	C14—H14	0.9500
C1—C6	1.398 (5)	C15—C16	1.381 (5)
C2—C3	1.380 (6)	C16—C17	1.410 (6)
C2—H2	0.9500	C16—H16	0.9500
C3—C4	1.367 (6)	C17—C18	1.336 (7)
C3—H3	0.9500	C17—H17	0.9500
C4—C5	1.394 (6)	C18—H18	0.9500
			
O1—S1—O2	119.6 (2)	C7—C8—N1	125.6 (3)
O1—S1—N1	108.15 (18)	N3—C9—C8	104.4 (3)
O2—S1—N1	107.40 (18)	N3—C9—C11	124.3 (3)
O1—S1—C1	106.36 (18)	C8—C9—C11	131.3 (4)
O2—S1—C1	109.6 (2)	N1—C10—H10A	109.5
N1—S1—C1	104.78 (18)	N1—C10—H10B	109.5
C18—S2—C15	90.7 (2)	H10A—C10—H10B	109.5
C8—N1—C10	117.9 (3)	N1—C10—H10C	109.5
C8—N1—S1	111.9 (2)	H10A—C10—H10C	109.5
C10—N1—S1	119.6 (3)	H10B—C10—H10C	109.5
C7—N2—N3	103.6 (3)	C9—C11—H11A	109.5
N2—N3—C9	113.7 (3)	C9—C11—H11B	109.5
N2—N3—C12	119.0 (3)	H11A—C11—H11B	109.5
C9—N3—C12	127.4 (3)	C9—C11—H11C	109.5
C13—N4—N5	119.4 (3)	H11A—C11—H11C	109.5
C13—N4—H4N	120.3	H11B—C11—H11C	109.5
N5—N4—H4N	120.3	N3—C12—C13	112.5 (3)
C14—N5—N4	115.1 (4)	N3—C12—H12A	109.1
C2—C1—C6	120.9 (4)	C13—C12—H12A	109.1
C2—C1—S1	119.7 (3)	N3—C12—H12B	109.1
C6—C1—S1	119.2 (3)	C13—C12—H12B	109.1
C3—C2—C1	119.3 (4)	H12A—C12—H12B	107.8
C3—C2—H2	120.3	O3—C13—N4	124.5 (4)
C1—C2—H2	120.3	O3—C13—C12	124.0 (4)
C4—C3—C2	120.6 (4)	N4—C13—C12	111.4 (3)
C4—C3—H3	119.7	N5—C14—C15	120.8 (4)
C2—C3—H3	119.7	N5—C14—H14	119.6
C3—C4—C5	120.5 (4)	C15—C14—H14	119.6
C3—C4—H4	119.7	C16—C15—C14	126.8 (4)
C5—C4—H4	119.7	C16—C15—S2	110.6 (3)
C6—C5—C4	119.7 (4)	C14—C15—S2	122.5 (3)
C6—C5—H5	120.1	C15—C16—C17	113.2 (4)
C4—C5—H5	120.1	C15—C16—H16	123.4
C5—C6—C1	118.7 (3)	C17—C16—H16	123.4
C5—C6—C7	123.5 (3)	C18—C17—C16	111.6 (4)
C1—C6—C7	117.7 (3)	C18—C17—H17	124.2
N2—C7—C8	111.9 (3)	C16—C17—H17	124.2
N2—C7—C6	124.4 (3)	C17—C18—S2	113.8 (4)
C8—C7—C6	123.7 (3)	C17—C18—H18	123.1
C9—C8—C7	106.4 (3)	S2—C18—H18	123.1
C9—C8—N1	127.8 (3)		
			
O1—S1—N1—C8	−68.6 (3)	N2—C7—C8—C9	0.3 (5)
O2—S1—N1—C8	161.0 (3)	C6—C7—C8—C9	178.8 (3)
C1—S1—N1—C8	44.5 (3)	N2—C7—C8—N1	−175.7 (4)
O1—S1—N1—C10	147.4 (3)	C6—C7—C8—N1	2.7 (6)
O2—S1—N1—C10	17.0 (4)	C10—N1—C8—C9	−63.6 (6)
C1—S1—N1—C10	−99.4 (3)	S1—N1—C8—C9	151.8 (4)
C7—N2—N3—C9	−1.2 (4)	C10—N1—C8—C7	111.6 (4)
C7—N2—N3—C12	178.5 (3)	S1—N1—C8—C7	−33.0 (5)
C13—N4—N5—C14	177.0 (3)	N2—N3—C9—C8	1.4 (4)
O1—S1—C1—C2	−98.9 (3)	C12—N3—C9—C8	−178.2 (3)
O2—S1—C1—C2	31.7 (4)	N2—N3—C9—C11	−178.9 (4)
N1—S1—C1—C2	146.7 (3)	C12—N3—C9—C11	1.4 (6)
O1—S1—C1—C6	77.1 (3)	C7—C8—C9—N3	−1.0 (4)
O2—S1—C1—C6	−152.2 (3)	N1—C8—C9—N3	174.9 (4)
N1—S1—C1—C6	−37.3 (3)	C7—C8—C9—C11	179.3 (4)
C6—C1—C2—C3	−2.7 (6)	N1—C8—C9—C11	−4.7 (7)
S1—C1—C2—C3	173.3 (3)	N2—N3—C12—C13	90.1 (4)
C1—C2—C3—C4	−1.6 (6)	C9—N3—C12—C13	−90.3 (5)
C2—C3—C4—C5	3.9 (7)	N5—N4—C13—O3	1.8 (6)
C3—C4—C5—C6	−1.9 (7)	N5—N4—C13—C12	−175.2 (3)
C4—C5—C6—C1	−2.3 (6)	N3—C12—C13—O3	22.4 (6)
C4—C5—C6—C7	174.3 (4)	N3—C12—C13—N4	−160.5 (3)
C2—C1—C6—C5	4.6 (6)	N4—N5—C14—C15	−179.9 (3)
S1—C1—C6—C5	−171.5 (3)	N5—C14—C15—C16	−179.7 (4)
C2—C1—C6—C7	−172.2 (4)	N5—C14—C15—S2	−3.7 (6)
S1—C1—C6—C7	11.8 (5)	C18—S2—C15—C16	0.3 (3)
N3—N2—C7—C8	0.5 (4)	C18—S2—C15—C14	−176.3 (4)
N3—N2—C7—C6	−177.9 (3)	C14—C15—C16—C17	176.3 (4)
C5—C6—C7—N2	10.2 (6)	S2—C15—C16—C17	−0.1 (5)
C1—C6—C7—N2	−173.2 (4)	C15—C16—C17—C18	−0.2 (6)
C5—C6—C7—C8	−168.0 (4)	C16—C17—C18—S2	0.5 (6)
C1—C6—C7—C8	8.6 (5)	C15—S2—C18—C17	−0.5 (4)

### Hydrogen-bond geometry (Å, °)

**Table:**

*D*—H···*A*	*D*—H	H···*A*	*D*···*A*	*D*—H···*A*
N4—H4N···O3^i^	0.88	2.10	2.964 (4)	166
C12—H12A···N5^i^	0.99	2.52	3.488 (5)	164
C11—H11B···O1^ii^	0.98	2.40	3.243 (6)	143

Symmetry codes: (i) −*x*+3/2, *y*, *z*+1/2; (ii) −*x*+2, −*y*, *z*+1/2.
